# Supplementary material for: Mycobacterium tuberculosis Fatty Acyl-CoA Synthetase fadD33 Promotes Bacillus Calmette–Guérin Survival in Hostile Extracellular and Intracellular Microenvironments in the Host
Source: Cells. 2023 Nov 11;12(22):2610. doi: 10.3390/cells12222610 (PMC10670722; doi:10.3390/cells12222610)
Supplement: Supplementary file 1 [file cells-12-02610-s001.zip › cells-2591140-supplementary.pdf]

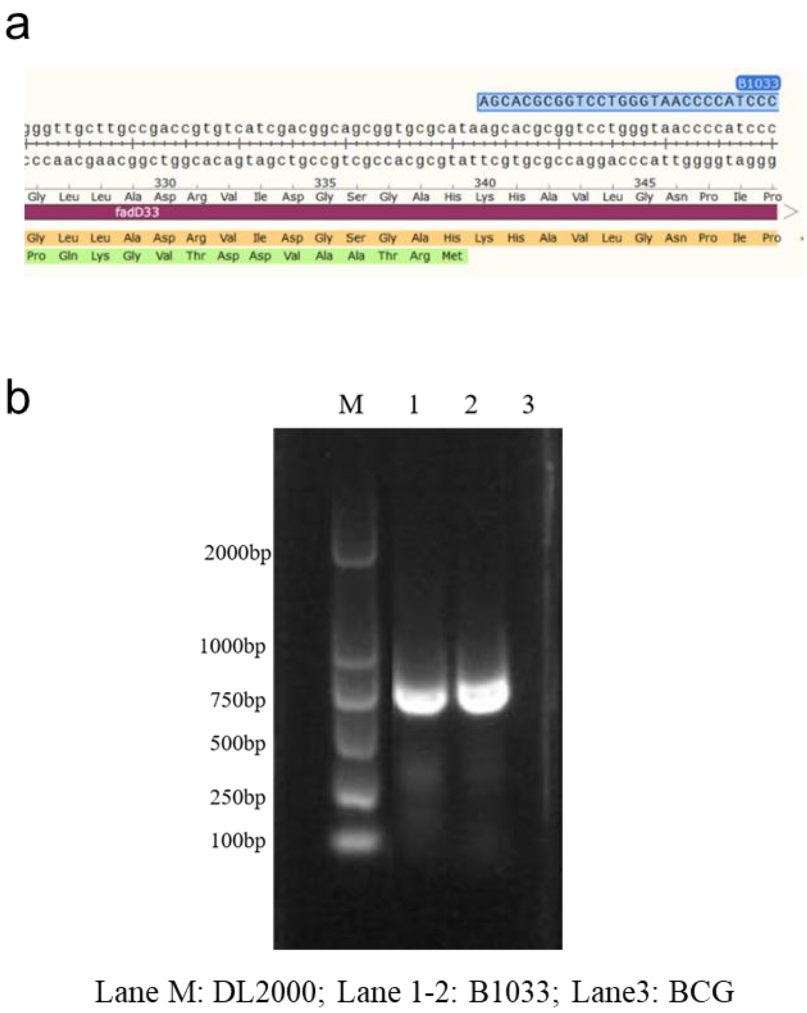

Figure S1. Insertion site verification of B1033 mutant. (**a**) insertion site map of B1033 mutant. The fragment size was 1566 bp, the transposon was inserted into the 1018 site of the gene. (**b**) PCR verification of recombinant B1033C strain.
